# Individual Identification of Prey in Carnivore Scats

**DOI:** 10.1111/1755-0998.70164

**Published:** 2026-06-23

**Authors:** Charlotte E. Eriksson, Marcus Bianco, Joel Ruprecht, Darren A. Clark, Taal Levi

**Affiliations:** ^1^ Department of Fisheries, Wildlife and Conservation Sciences Oregon State University Corvallis Oregon USA; ^2^ Oregon Department of Fish and Wildlife La Grande Oregon USA

**Keywords:** amplicon sequencing, environmental DNA, fecal DNA, noninvasive genetic sampling, scats, single nucleotide polymorphism

## Abstract

Noninvasive genetic sampling is widely used in ecology and conservation to identify predators and their diets but recovering individual‐level information from consumed prey remains largely unexplored. We evaluated whether individual prey can be reliably genotyped from carnivore scats and assessed limitations associated with degraded and mixed DNA sources. We developed a 31‐locus SNP panel optimized for genotyping degraded elk ( *Cervus canadensis* ) DNA using amplicon sequencing. We validated prey genotyping by matching elk genotypes recovered from carnivore scats (‘carcass scats’) collected at cougar ( *Puma concolor* ) kill sites to genotypes from corresponding elk carcasses. We also genotyped scats collected throughout the study area, identified as containing elk using DNA metabarcoding (‘survey scats’). To avoid misidentifying multiple individuals in a scat as a unique genotype, we evaluated artificial mixtures of prey DNA to assess the ability to detect and filter samples containing mixed DNA. Elk genotypes recovered from carcass scats matched associated carcass genotypes, confirming accurate recovery of individual prey DNA from carnivore scats. Genotyping success was 88% in fresh carcass scats and 74% in survey scats of unknown age. Heterozygosity excess filtering removed most mixed samples, although one of 24 mixtures with equal DNA contributions from two individuals produced a false unique genotype. Our results demonstrate that carnivore scats can serve as a reliable source of individual‐level prey DNA under appropriate conditions. This method provides a new data stream on individual prey mortality, predation rates, and scavenging dynamics, processes that have previously been difficult to quantify without invasive capture and collaring techniques.

## 1 Introduction

Noninvasive genetic sampling has become an essential tool in ecology and conservation by providing valuable data on wildlife without the need for capture or direct observation. Among the various types of noninvasive samples, scats offer the most information including individual identification, population genetics and demographics, microbiome, parasites, hormones and more (Guo et al. 2021; Kersey and Dehnhard 2014; Srivathsan et al. 2016). While most of these genetic data relate to the ‘host’ (i.e., the animal that deposited the scat), carnivore scats also contain mitochondrial DNA from prey species, commonly targeted using DNA metabarcoding in diet studies (e.g., Eriksson et al. 2024; Tosa et al. 2023). However, carnivore scats also contain nuclear DNA from consumed prey, although typically at lower concentrations. If this nuclear DNA can be reliably genotyped, it opens a new and largely untapped source of individual‐level information about prey species. Such data could expand our ability to infer prey mortality, track fates of known individuals, estimate predator impact (by quantifying the number of individuals consumed, not just species presence), and study scavenging dynamics. It could also enable new applications of statistical models such as spatial capture‐recapture (SECR) models for density estimation (Borchers and Efford 2008) applied to prey genotypes found in scats, direct estimation of predator kill rates, or integrated population models (IPMs) that include genetically‐confirmed predation events.

Despite its promise, several biological and technical challenges complicate genetic sampling of prey within predator scats. Scats are dominated by bacterial and host DNA (Perry et al. 2010), while prey DNA is often present in low quality and quantity. A single scat may also contain mixed DNA from several prey individuals, which can confound genotype assignment. The feasibility of individual prey genotyping is thus likely to vary depending on predator foraging behaviour and prey size. For instance, predators that typically consume single large prey items (e.g., cougars *Puma concolor* ) are more likely to produce scats with DNA from a single individual, whereas those that feed on small or aggregated prey (e.g., foxes, mustelids or bears foraging on salmon) or routinely scavenge (e.g., canids) may yield complex DNA mixtures. However, if allele frequencies of the prey population are known, the number of contributors can be estimated (Andres et al. 2021; Shi et al. 2023), and mixed samples can be identified and filtered, reducing the risk of false individual assignments.

To our knowledge, individual‐level genotyping of consumed prey from carnivore scats has not been successfully demonstrated. However, recent advances in molecular techniques including next‐generation sequencing and SNP‐based genotyping have greatly improved the sensitivity of noninvasive genetic analyses (Carroll et al. 2018; Eriksson et al. 2020), potentially allowing for efficient detection of individual prey genotypes in carnivore scats.

In this study, we explore whether consumed prey can be reliably genotyped from carnivore scats using genotyping by amplicon sequencing. Single nucleotide polymorphisms (SNPs) are increasingly preferred over microsatellites for genotyping degraded DNA due to their compatibility with short amplicon sequencing and improved genotyping success in noninvasive samples (Fabbri et al. 2012; von Thaden et al. 2017). Because fecal DNA is often highly degraded, markers producing short amplicons are expected to reduce allelic dropout and improve genotyping success relative to microsatellite loci. We therefore developed a SNP panel for individual identification of elk ( *Cervus canadensis* ) from degraded samples, validated prey genotyping by matching elk genotypes from carnivore scats collected at cougar kill sites to genotypes from the corresponding elk carcasses, and applied our method to a larger set of field‐collected scats to assess applicability in a natural setting. We also experimentally tested artificial mixtures of prey DNA, from two to four individuals with either equal or varying input concentrations, to evaluate how well mixed genotypes could be detected and filtered out.

## 2 Materials and Methods

### 2.1 Study Area

**FIGURE 1:**
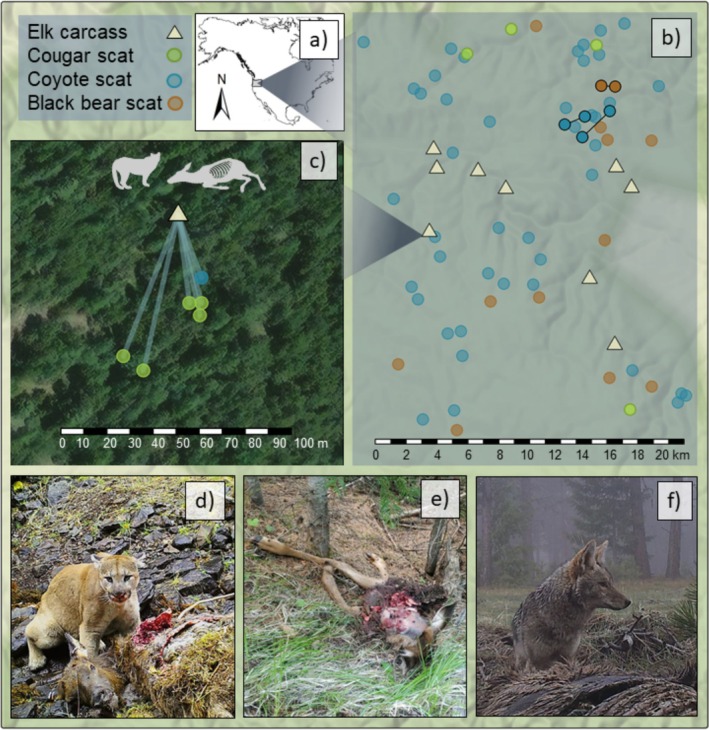
(a) Study area in northeastern Oregon, USA. (b) Map of successfully genotyped survey scats collected using scat detection dogs in 2017, and elk carcasses located in 2022–2023. A small amount of jitter was added so the scat locations did not fully overlap. Two carcasses were located outside of Starkey Experimental Forest and are not depicted on the map. Recaptured elk genotypes in two pairs of coyote scats indicated by connecting line, and two black bear scats. (c) Example of elk carcass and surrounding carnivore scats containing elk DNA matching the associated carcass genotype. (d) Cougar at an elk carcass. (e) Elk carcass at a cougar kill site (f) coyote scavenging a carcass.

All samples were sourced from Starkey Experimental Forest and Range and adjacent public lands in the Blue Mountains in northeastern Oregon, USA (Figure 1a). Starkey is enclosed by a 2.4‐m fence that spans approximately 100 km^2^ and restricts movement of large herbivores like elk, while still allowing carnivores and smaller non‐ungulate species to move freely (Rowland et al. 1997; Ruprecht et al. 2021a). Carnivore species in the area include cougars, black bears ( *Ursus americanus* ), bobcats ( *Lynx rufus* ), coyotes ( *Canis latrans* ) and occasionally gray wolves ( *Canis lupus* ). Large herbivores include elk, mule deer ( *Odocoileus hemionus* ) and white‐tailed deer ( *O. virginianus* ).

### 2.2 Sample Types and DNA Extraction

#### 2.2.1 SNP Panel Development Samples

In winter 2020, whole blood (2 mL) was sampled from 19 adult female elk during live‐captures and stored in EDTA tubes. Additionally, tissue samples from three male elk were obtained from hunted individuals within the wider Blue Mountains region and stored frozen until DNA extraction. DNA was extracted from the tissue and blood samples using the DNeasy Blood & Tissue kit following the manufacturer's protocol (Qiagen, Hilden, Germany). All animal handling procedures were approved by the USDA Forest Service, Starkey Experimental Forest Institutional Animal Care and Use Committee (IACUC No. 92‐F‐0004) and followed protocols described in Wisdom et al. (1993).

#### 2.2.2 Carcass Samples

Cougars were GPS‐collared as part of a larger study conducted in 2022–2023 (ODFW unpublished data). Kill sites were identified based on GPS clusters and visited within 7.25 ± 4.37 SD days (min 2, max 16) to locate elk carcasses. Tissue or hair samples were collected from carcasses and stored frozen or in desiccant (hair). Any carnivore scats found within 200 m of the carcass were collected and dried in a drying oven at 40°C for 24 h. Carcass tissue and hair samples were extracted as described above. For scat samples, we subsampled three small pieces per scat into a 2 mL Eppendorf tube (approximately 0.25 g in total) and extracted DNA using the DNeasy Blood & Tissue kit (Qiagen) with the following modifications: 550 μL buffer ATL, 50 μL Proteinase K and a 4‐h incubation step.

#### 2.2.3 Survey Scats

We used carnivore scat DNA extracts used in previous studies (Ruprecht et al. 2021a, 2021b). Scats were collected in Starkey using detection dogs in early summer 2017, overlapping the elk parturition period. Scats were stored dried in paper bags until extraction within 3 months later. From these, we selected 93 scats with the highest number of elk reads based on 12S metabarcoding results (Ruprecht et al. 2021a, 2021b), as higher read counts were expected to correlate with higher elk DNA concentrations, potentially improving genotyping success.

#### 2.2.4 Artificially Mixed Samples

To evaluate whether mixed DNA samples could be identified and excluded, we constructed 24 artificial mixtures using six randomly selected blood samples from individuals previously used in the SNP panel development. These mixtures combined DNA from two, three, or four individuals at varying concentrations and were designed to explore a range of potential mixed‐prey DNA profiles that might occur in carnivore scats, given variation in prey size, digestion time and foraging behaviour (Table 1). Mixture Type 1 involved individuals contributing equal amounts of DNA, representing a case where a carnivore had consumed two, three, or four different prey individuals within the gut‐passage window. Mixture Type 2 was the same as the first but with lower DNA concentrations (0.5 ng per individual as opposed to 2 ng) to simulate a more degraded scat. Mixture Type 3 simulated an unbalanced mixture, with one dominant individual contributing 2 ng of DNA and others contributing 1 ng each. Mixture Type 4 included three individuals with decreasing DNA input (0.5 ng, 0.25 ng and 0.125 ng), representing a degraded scat with minor contributions from three individuals. Finally, Mixture Type 5 consisted of a single dominant individual (2 ng) and a trace contribution from a second individual (0.125 ng) (Table 1). This last type may reflect the most common biological scenario, as we lack precise information on how long prey DNA persists in a carnivore's digestive system. Trace DNA from a previously consumed prey item may still be present when a new prey is ingested. The DNA concentration of each tissue extract was quantified using a Qubit 2.0 fluorometer (Life Technologies) with the AccuGreen High Sensitivity dsDNA Quantitation Kit (Biotium). Each extract was diluted to 2 ng DNA in 20 μL, from which a series of two‐fold serial dilutions (1, 0.5, 0.25 and 0.125 ng in 20 μL) was prepared. Each mixture was prepared to a final volume of 10 μL using nuclease‐free water, and 1 μL was used as template per genotyping PCR reaction, as described below.

**TABLE 1:** Artificial DNA mixtures used to evaluate the detection of mixed prey genotypes in carnivore scats.

*N* individuals	Mix input type	DNA input (ng)
2	Mixture type 1	2, 2
2	Mixture type 1	2, 2
2	Mixture type 1	2, 2
3	Mixture type 1	2, 2, 2
3	Mixture type 1	2, 2, 2
3	Mixture type 1	2, 2, 2
4	Mixture type 1	2, 2, 2, 2
4	Mixture type 1	2, 2, 2, 2
4	Mixture type 1	2, 2, 2, 2
2	Mixture type 2	0.5, 0.5
2	Mixture type 2	0.5, 0.5
2	Mixture type 2	0.5, 0.5
2	Mixture type 3	2, 1
2	Mixture type 3	2, 1
2	Mixture type 3	2, 1
3	Mixture type 3	2, 1, 1
3	Mixture type 3	2, 1, 1
3	Mixture type 3	2, 1, 1
3	Mixture type 4	0.5, 0.25, 0.125
3	Mixture type 4	0.5, 0.25, 0.125
3	Mixture type 4	0.5, 0.25, 0.125
2	Mixture type 5	2, 0.125
2	Mixture type 5	2, 0.125
2	Mixture type 5	2, 0.125

*Note:* Each mixture combined DNA from 2 to 4 elk individuals at varying input amounts, following five predefined mixture types (see Methods for detailed descriptions). ‘DNA input’ indicates the amount of DNA contributed by each individual elk sample in the mixture. Mixture Type 1 = equal input, Type 2 = equal but low input, Type 3 = unbalanced input, Type 4 = degraded low inputs, Type 5 = one dominant, one trace contributor.

#### 2.2.5 Elk SNP Panel

We created a new elk SNP panel for genotyping by amplicon sequencing of degraded DNA based on SNP positions and flanking regions found in a microfluidics panel for tule elk (*Cervus canadensis nannodes*) (Sacks et al. 2021). We evaluated a set of 35 candidate SNPs for which primers could be successfully designed according to guidelines in Eriksson et al. (2020), which emphasize minimizing primer interactions and standardizing annealing temperatures for multiplex PCR. A smaller panel helps reduce primer dimer formation, which is especially important when working with degraded or low‐quantity DNA, while still providing sufficient resolution for individual identification. We also designed primers amplifying the sex determining SRY region using the *Cervus canadensis roosevelti* reference sequence (GenBank accession DQ888686.1) as a template for primer design. The primers were appended with Illumina P7 and P5 overhang adapter sequences and ordered in 100 μM concentrations using standard desalting purification from Integrated DNA Technologies. All primers were checked for amplification, product size, and annealing temperature and initial primer concentration optimization in singleplex PCR reactions and visualized on a 1% agarose gel. The loci were assessed for polymorphism in elk from our study area by genotyping the 22 blood and tissue samples following the methods described by Eriksson et al. (2020). Briefly, all primers were amplified in multiplex PCR reactions, the PCR products were purified with magnetic beads and then used as template in a second PCR reaction where index barcodes were added to allow for sample identification after pooling. The samples were then quantified, normalized, and pooled per 96‐well plate. Each plate pool was then purified, quantified and normalized before pooled into one tube and submitted for 150 bp sequencing on a HiSeq 3000 at the Center for Quantitative Life Sciences at Oregon State University. We estimated the probability of identity (PID) and probability of identity among siblings (PIDsibs) based on the tissue and blood samples using GenAlEx v 6.5 (Peakall and Smouse 2012). We also tested for cross‐species amplification by genotyping carnivore tissue samples (coyote, black bear, cougar) and other herbivores (white tailed deer and mule deer) present in the area.

#### 2.2.6 Carnivore and Prey Species Identification With DNA Metabarcoding

We identified prey and carnivore DNA in the carcass scats using DNA metabarcoding following methods described previously (Ruprecht et al. 2021a, 2021b). Briefly, each sample was amplified with slightly modified vertebrate primers 12SV5F and 12SV5R (used in Eriksson et al. 2019; adapted from Riaz et al. 2011), in 20 μL PCR reactions consisting of 10 μL Amplitaq Gold Master mix (ThermoFisher Scientific, Waltham, MA, USA), 3 μL of water, 5 μL primer mix (0.25 μM final concentration) and 2 μL DNA template. Each PCR reaction was amplified with identical unique 8 bp tags on the 5′ end of the forward and reverse primers for downstream sample identification and to prevent tag jumping (Schnell et al. 2015). Each sample was amplified in triplicate and we included three no‐template controls per 96‐well plate. Library prep, sequencing and bioinformatic methods have been described previously (Ruprecht et al. 2021a, 2021b).

#### 2.2.7 Individual Identification of Carnivore and Prey

All samples were genotyped using the new elk SNP panel using methods described above. PCRs were set up in HEPA‐filtered and UV‐irradiated PCR cabinets within a pre‐PCR laboratory. Scat and tissue samples were processed in different PCR cabinets using separate pipettes to prevent contamination of the lower quality DNA samples.

The raw genotyping reads were demultiplexed using bcl2fastq (Illumina). We called genotypes using the GTseq PERL scripts by Campbell et al. (2015) which counts the number of reads containing each allele and assigns a genotype based on the ratio of allele 1 to allele 2. A ratio of > 10 was called homozygous for allele 1, < 0.1 homozygous for allele 2, and < 2 were called heterozygous (Campbell et al. 2015). Correspondingly, allele ratios ≥ 2 and ≤ 10 and ≥ 0.1 and ≤ 0.2 were considered ambiguous and left unassigned. We used the three replicates to construct a consensus genotype by requiring two replicates to call a heterozygous genotype and all three replicates for a homozygous genotype. Samples that produced a consensus genotype at fewer than 24 loci (< 77% amplification success) were considered to have ‘failed’ and were excluded from further analysis. This threshold was chosen because genotypes with ≥ 24 loci yielded a probability of identity (PID) of 2.3 × 10^−8^ and a probability of identity among siblings (PIDsibs) of 1.19 × 10^−4^, providing sufficient power for individual discrimination. We used the R package *allelematch* to check for matches of elk genotypes in multiple carnivore scats (Galpern et al. 2012). Genotypes were considered matches if they differed at two or fewer loci.

To identify and remove mixed samples, we evaluated two filtering methods using heterozygosity excess and missing loci thresholds. For the first method, we calculated observed heterozygosity (Ho) per sample as the proportion of heterozygous loci among all successfully genotyped loci and compared it to expected heterozygosity (He) derived from allele frequencies in the single‐individual tissue or blood samples used for SNP development (*n* = 22, mean He = 0.35, SD = 0.13). We then quantified heterozygosity excess as Ho—He for each sample. Mixed samples are expected to show elevated heterozygosity due to the presence of alleles from multiple individuals. Based on the distribution of heterozygosity excess in single individuals (max 0.13, mean −0.01 ± 0.09), we set the heterozygosity‐excess threshold above this observed maximum (< 0.18) because that value maximized identification of mixed samples but still maintained a low false positive rate. A threshold of 0.18 corresponded to an estimated false‐positive rate of 1.73%, such that fewer than 2% of true single‐individual samples would be incorrectly classified as mixed. The second filtering method we considered was based on a threshold for the number of loci that failed to amplify. Mixed samples are unlikely to conform to the expected allele ratios used by the GTseq pipeline to assign genotypes, resulting in ambiguous intermediate allele ratios that fall outside the genotype‐calling thresholds (i.e., ratios between ≥ 2 and ≤ 10) and are therefore recorded as missing (‘NA’). Consequently, mixed samples were expected to be filtered out due to high proportions of missing loci. This second filtering method may be an option in systems where allele frequencies of the prey population are unknown. Because allelic dropout associated with degraded DNA is expected to reduce observed heterozygosity while increasing missing data rather than inflate heterozygosity, mixed samples that are also highly degraded may not exceed the heterozygosity‐excess threshold and are instead removed by the missingness filter.

## 3 Results

### 3.1 Elk SNP Panel

We initially tested 35 SNPs, of which 32 were polymorphic. Aside from the three monomorphic loci, one was removed because it amplified carnivore species, one was not unique within the elk genome and one for causing primer dimers with other primers. We re‐designed 5 primers to minimize primer dimers. Three of the loci contained a second, unlinked SNP, bringing the total number of SNPs to 31. Overall the SNP panel had a PID value of 5 × 10^−10^ and PIDsibs 1.7 × 10^−5^. The SRY locus accurately assigned sex to all tissue and blood samples. 25 out of the 31 loci also amplified the two deer species, necessitating exclusion of scat samples that contain both elk and deer to avoid misassigning deer DNA as falsely unique elk genotypes.

### 3.2 Detection of Mixed DNA Using Heterozygosity and Missingness

**FIGURE 2:**
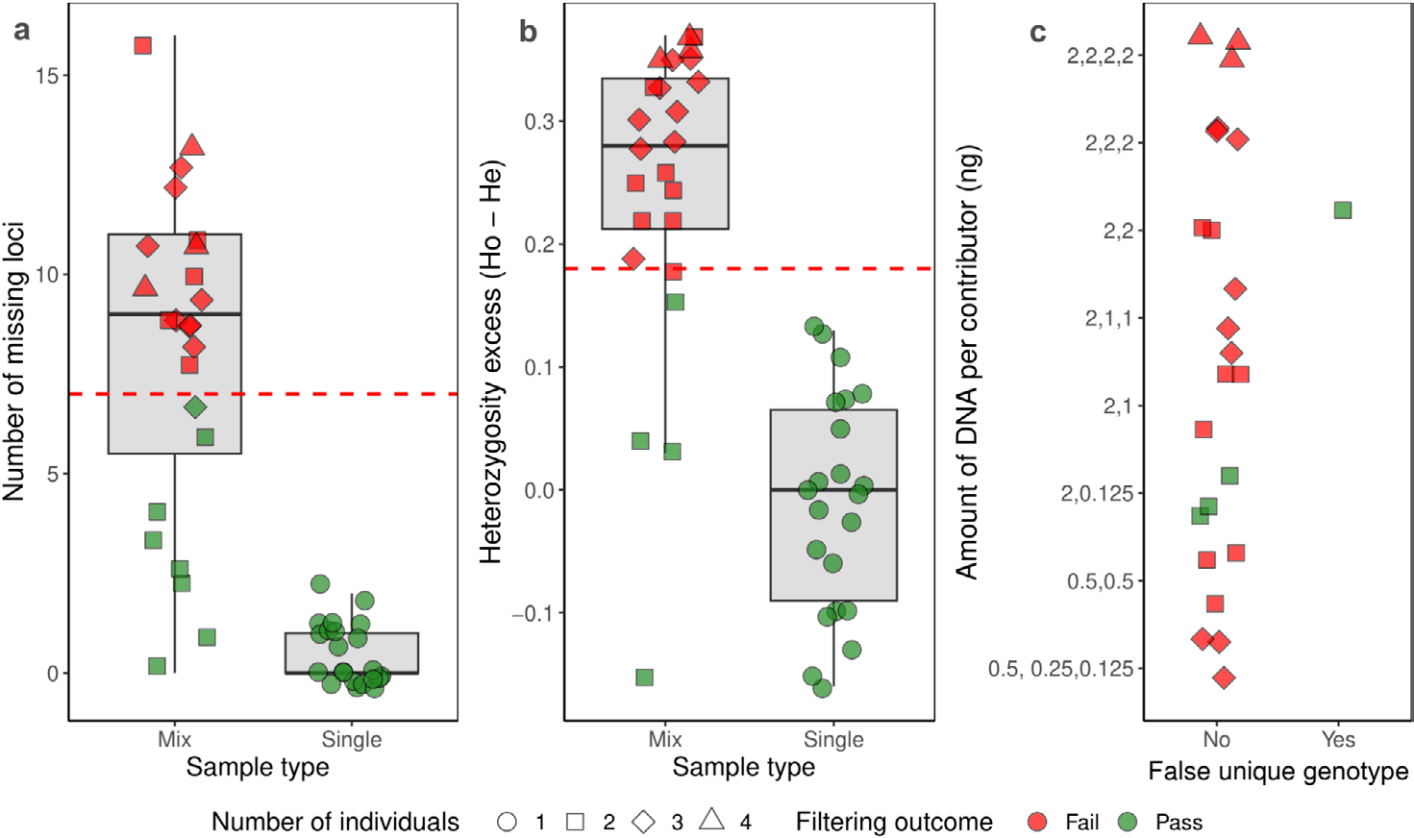
Filtering methods used to evaluate detection of mixed prey DNA in artificial mixtures. (a) Number of missing loci in mixed versus single individual DNA samples. Red dashed line represents filtering threshold for number of missing loci (7 out of 31 loci). (b) Heterozygosity excess in mixed compared to single contributor samples. Red dashed line represents filtering threshold (0.18). Colours indicate whether a sample failed or passed filtering. (c) Four mixed samples passed the heterozygosity excess filtering threshold, but only one resulted in a false unique genotype. The other three samples that passed filtering resulted in only the major contributor's genotype, suggesting that samples with minor input from a second individual will not result in a mixed genotype.

Heterozygosity excess was a better filtering criterion for mixed samples with 20 out 24 artificial mixtures successfully filtered compared to 16 using the missingness filter (Figure 2a,b). Four mixed samples passed the heterozygosity excess threshold. Three of these represented uneven DNA inputs where one individual dominated the sample (Mixture type 3 and 5). The resulting genotype for those samples matched the dominating individual and would therefore not introduce a false unique genotype to the final results. However, the fourth sample represented two equally contributing individuals with a resulting mixed genotype that falsely appeared as a unique individual (Figure 2c). This resulted in 1 of 24 DNA mixtures being misinterpreted as a unique individual.

### 3.3 Carcass Samples

We located 11 elk carcasses based on cougar GPS clusters and sampled tissue (*n* = 10) or hair (*n* = 1). All carcass tissue and hair samples genotyped successfully. A total of 21 scat samples were collected around the carcasses, of which 16 contained elk DNA (two failed metabarcoding and three did not contain elk). Among these 16 scats, two were from black bears, three from coyotes, and 11 from cougars. Every carcass had at least one scat with a perfectly matching elk genotype, validating that prey DNA can be reliably recovered from carnivore scats. Notably, one female elk that we obtained a blood sample from in 2020 for SNP panel development was identified through genotyping as one of the carcasses and was detected in a carcass scat from a cougar in 2022, highlighting the ability of this approach to determine fate or cause of mortality of known individuals.

Across all kill sites except one, a single elk genotype was detected in scats associated with each carcass. At the remaining kill site, three unique elk genotypes were identified across six scat samples. One female elk was found in three scats (1 coyote and 2 cougar) and matched the genotype from the associated carcass. Two additional cougar scats contained two unique male elk genotypes. A sixth sample produced a genotype consistent with a mixture of the carcass‐associated female and one of the male individuals. This sample exhibited heterozygous genotypes at several loci where the other two individuals were homozygous for opposite alleles. It also lacked any unique alleles not present in the other two, had an ambiguous sex assignment, and was ultimately filtered out due to elevated heterozygosity. The fourth sample represented a third, genetically distinct elk genotype with several unique alleles.

### 3.4 Elk Genotyping Success in Carnivore Scats

The 93 survey scats came from coyotes (*n* = 73), black bears (*n* = 14), cougars (*n* = 4), and two contained no carnivore DNA that passed metabarcoding filtering thresholds. After excluding six scats that contained both deer and elk, 61 of the survey scats and 15 of the carcass scats passed the missingness filter (74% and 88% genotyping success, respectively). Of these, 7 survey scats (6 coyote scats and one scat without predator ID) and 1 carcass scat (described above) showed heterozygosity excess. The remaining 54 survey scat samples contained 51 unique elk individuals. There were two individual elk found in two coyote scats each and one elk found in two bear scats (Figure 1b). Recaptured individuals were found in scats between 43 m and 1723 m apart (mean 917 m).

## 4 Discussion

This study demonstrates the feasibility of genotyping individual prey from carnivore scats using a new SNP panel and amplicon sequencing. While noninvasive genetic methods have traditionally focused on the host species (e.g., Waits and Paetkau 2005; von Thaden et al. 2017), our results show that carnivore scats can also be a reliable source of individual‐level DNA from consumed prey. We achieved a 74% genotyping success rate in the survey scats and an 88% success rate in the carcass scats from known cougar kill sites. Elk genotypes recovered from carcass scats matched those of nearby carcasses, providing validation that prey DNA can be recovered and accurately assigned.

The ability to identify individual prey in carnivore scats expands the utility of noninvasive genetic sampling to questions involving prey mortality, individual fates, and predator–prey interactions. When the prey population is independently genotyped, this approach can detect mortality of known individuals and reveal predator selection for specific age classes or sexes, enhancing demographic estimates without the need for resource‐intensive capture or GPS collaring. In highly endangered prey populations, this method could also be a more efficient and safe way of determining cause‐specific mortality.

Importantly, prey genotyping from carnivore scats represents a new data stream for studying predation that enables direct, noninvasive estimation of predation impacts by identifying the number of prey individuals consumed, rather than relying on species‐level frequency of occurrence metrics, which may overestimate predation rates when multiple scats originate from a single prey item. We exemplify this with the same elk individuals found across multiple scats from different carnivores. In addition, linking individual prey to predator species could reveal whether a predator is actively killing or scavenging. For example, it could be used to determine if black bears mostly kill their own elk calves or primarily scavenge kills made by cougars, providing data that are very challenging to obtain in practice using current methods.

By recovering individual prey genotypes from carnivore scats, the number of prey individuals consumed within a defined spatial and temporal window can be estimated noninvasively. When combined with independent estimates of prey density, these data can be used to estimate predation rates (the proportion of the prey population killed per unit time). When combined with predator density, they can be used to estimate kill rates (the number of prey killed per predator per unit time). Because prey genotypes can be spatially referenced, they could also be incorporated into SECR‐type models (Kéry et al. 2011) to estimate predation impacts across the landscape. Thus, prey genotyping from scats provides the individual‐level mortality data that previously necessitated expensive and invasive animal capture and collaring methods. However, translating these detections into demographic rates will require models that explicitly account for the spatial and temporal window over which scats are detectable.

We identified 51 unique elk genotypes across 54 survey scats, with three individuals appearing in two samples each. Ruprecht et al. (2021b) showed that elk are the dominant prey of cougars in the area, that coyotes frequently scavenge cougar kills, and that black bears both scavenge and prey on elk calves. In this context, the relatively high number of unique individuals likely reflects elevated predation pressure during the elk parturition period, when neonates are particularly vulnerable and carnivore predation on elk is concentrated (Huggler et al. 2023; Ruprecht et al. 2022). Further, we used scats sampled across the area to meet objectives for other studies (Ruprecht et al. 2021a, 2021b), which may have further contributed to the high number of unique individuals detected. If the primary goal is to recapture individual prey, concentrating sampling effort within a smaller area would likely increase the probability of resampling the same individuals.

Our results further show that samples with mixed prey DNA can be filtered out in most cases due to either genotype dropout (missing data) or excess heterozygosity, with the two filtering approaches complementing each other. Artificial mixture tests demonstrated that nearly all mixed samples (23 out of 24; 96%) were either effectively filtered (20 out of 24; 83.3%) or matched the single dominant contributor (3 individuals in a mixture with 2 ng vs. 0.125 ng DNA). Importantly, using the missingness filter alone would have removed only 16 out of 24 artificial mixtures, highlighting the importance of incorporating heterozygosity‐based filtering. One mixture with equal DNA contributions (2 ng each) generated a false unique genotype that would inflate estimates of the number of prey individuals. This could be problematic in systems where predators frequently consume multiple individual prey in a short time frame. Accurate estimation of allele frequencies in the prey population is therefore critical for this method to reliably detect and filter mixed samples. We encourage researchers adopting this method to consider the natural history of the predator or scavenger species sampled to assess the likelihood they feed on multiple prey individuals within a single digestive passage. For example, cougar digestive passage time has been estimated between 1 to 4 days (Baune et al. 2021) while average prey search times (time between leaving one kill and making the next) have been reported at 4.9 days (summer) to 6.6 days (winter) (Knopff et al. 2010), suggesting only a modest likelihood of observing multiple individuals in a given scat. Future research should also quantify the detection window of prey DNA in carnivore scats post consumption, ideally through controlled feeding trials.

Scats collected at one of our 11 cougar kill sites revealed three distinct elk genotypes across six scats. One possibility is that this cougar killed and consumed several elk calves during her 3.5‐day residency at the site, which is consistent with documented cougar behaviour, as cougars may kill and feed on multiple neonate ungulates in close proximity (i.e., multiple individuals at elk nursery sites or sibling deer fawns) or consume multiple newborn individuals over consecutive days (Clark et al. 2014; Knopff et al. 2010). Alternatively, some of the elk DNA detected may derive from prey consumed prior to arrival at the kill site. The occurrence of a mixed prey genotype in one scat further supports simultaneous or sequential feeding on multiple calves. This is most likely to occur shortly after ungulate parturition when handling and consumption times for predators are short (Logan Bates‐Mundell et al. 2024); as prey increase in size and handling times are longer, this occurrence should decline. Overall, these results indicate that prey genotyping from scats is most reliable when identifying known individuals or in contexts such as ours, where multiple‐prey events appear to be rare (1 out of 11 kill sites).

Employing a larger SNP or microhaplotype panel than used here could enable the use of DNA mixture models to estimate the number of contributing individuals (Sethi et al. 2019; Shi et al. 2023), an approach previously applied to high‐quality DNA sources such as tissue. Simulations using SNP panels (64–192 loci) indicated improved robustness to genotyping error, but resolving mixtures of related individuals remains difficult (Sethi et al. 2019). These findings suggest that larger panels would be needed to reliably resolve complex mixtures. For example, in forensic applications, high‐density SNP microarrays (hundreds to thousands of loci) can accurately identify individual contributors even when they represent less than 0.1% of the total DNA in a mixture (Homer et al. 2008). However, microarrays are expensive and require specialized equipment often not available in wildlife labs. Microhaplotypes, which capture multiple linked SNPs within a single locus, could increase discriminatory power while keeping panel size manageable. Overall, these studies highlight the potential benefits of increasing panel size in systems where mixed prey DNA is likely. However, multiplexing large numbers of markers remains challenging with degraded DNA, and experimental validation in noninvasive samples is therefore an important next step. Importantly, both SNP panel size and heterozygosity‐based filtering thresholds are expected to be system‐specific and should be empirically calibrated for each study population based on its genetic diversity and allele frequency distribution. If genotyping success remains high with a larger SNP or microhaplotype panel, future studies could go beyond detecting the number of prey individuals in scats. Microhaplotypes could provide sufficient resolution to assess relatedness among prey individuals, potentially linking calves in scats to known adult females or identifying siblings. If combined with a temporal series of samples, this could enable reconstruction of family groups and insights into reproductive success or calf survival over time. Such analyses could also inform predator–prey dynamics, for instance by revealing whether certain individuals are preferentially predated. For species such as cougars, an alternative is to avoid sampling during the ungulate parturition period, when the likelihood of multiple ungulate kills within a shorter timeframe is elevated (Clark et al. 2014). However, in our study, mixed prey genotypes were rare (1 out of 11 kill sites), suggesting that the impact of sampling during parturition may be limited in this system. The extent to which genotype mixture prevalence influences study design is likely to be species‐ and system‐specific.

Our results demonstrate that carnivore scats can serve as a reliable source of individual‐level DNA from consumed prey under appropriate conditions. By enabling individual prey identification, this approach expands noninvasive genetic sampling beyond species detection to individual‐level inference, allowing predation events to be linked directly to specific prey. In monitored populations with known genotypes, prey genotyping from scats can reveal individual fates, detect otherwise undocumented mortality events, and refine estimates of predator impacts without the need for invasive capture or GPS collaring. With careful SNP panel development, stringent filtering, and strategic application, prey genotyping from carnivore scats represents a powerful addition to noninvasive genetic tools and opens new opportunities to study predation, scavenging networks, and prey demography.

## Author Contributions

C.E.E. and T.L. conceived the research; C.E.E. designed the methodology, performed assay design, laboratory work and analysis. J.R. and M.B. collected the samples; T.L. and D.C. led funding acquisition; C.E.E. led the writing of the manuscript. All authors contributed critically to the drafts and gave final approval for publication.

## Funding

This work was supported by the Oregon Department of Fish and Wildlife. National Science Foundation (2317537).

## Conflicts of Interest

The authors declare no conflicts of interest.

## Data Availability

Data and code required to reproduce the analyses and figures in this paper are available in Dryad at https://doi.org/10.5061/dryad.70rxwdccs.
